# Does retail investors beat institutional investors?——Explanation of game stop’s stock price anomalies

**DOI:** 10.1371/journal.pone.0268387

**Published:** 2022-10-25

**Authors:** Bin Gao, Huanhuan Hao, Jun Xie

**Affiliations:** 1 School of Economics, Guangxi University for nationalities, Nanning, China; 2 School of Economics, Guangxi University, Nanning, China; University of Almeria, SPAIN

## Abstract

This paper studies the relation of information cost, retail investor sentiment and asset pricing. Our motivation to study this model is to learn why retail investors could move asset price away from fundamental values. In the model, the institutional investors are pessimistic and the retail investors are optimistic, the ratio of the expected utility of informed and rational but uninformed institutional investors increases first and then decreases as the cost of information increases. In addition, a large number of retail investors promoted substantial increases in stock prices. This model provides part of the explanation for the unusually high stock price of Game Stop in early 2021 that retail investors cliqued and confronted institutional investors.

## 1. Introduction

**Fig 1:**
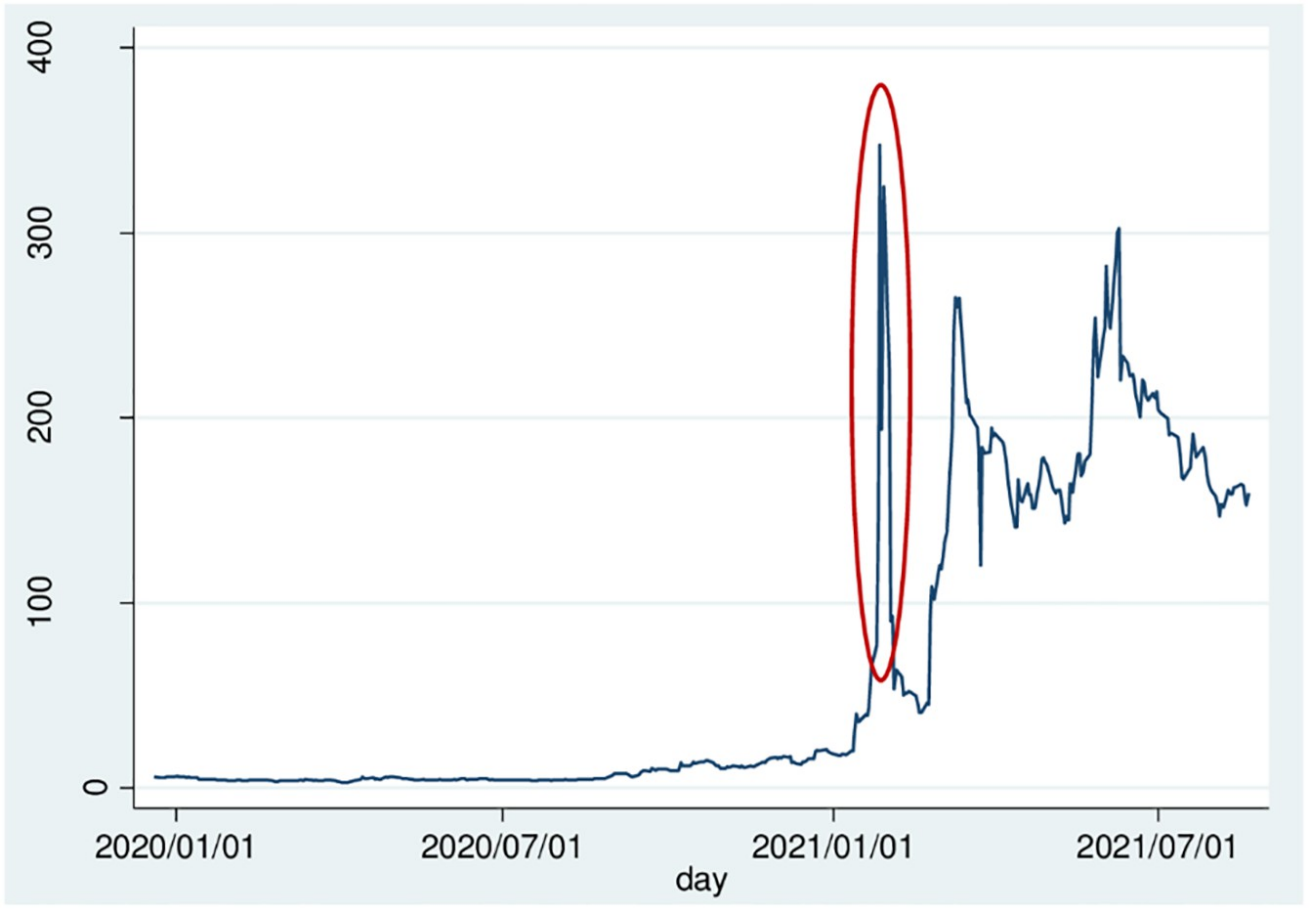
Game Stop’s closing price trend chart from December 2019 to August 2021.

According to Hong et al. [1, 2], they conducted a cutting-edge research on oral information dissemination represented by some social relations, such as neighborhood and community relations, and classmate relations, and found that the social interaction has a significant impact on the degree of investors’ participation in stock market trading activities. In recent years, due to the popularization of the Internet and the development of financial technology, the cost of information required for investors to conduct transactions has been reduced, and the efficiency of interpersonal information transmission has also been enhanced. In addition, compared with institutional investors, retail investors have limited information, technology, and funds, and are more susceptible to sentiment. Therefore, in the Chinese stock market, the trading behavior of retail investors, who account for a mass of trading volume, will undoubtedly have a significant impact on the market. Motivated by these results, in this article, based on the research framework of Grossman and Stiglitz [3], we construct an asset pricing model about information cost and investor sentiment to study how retail investors clique behavior affects asset prices. It provides some explanations for the 2021 Game Stop event. (As shown in Fig 1).

Traditional financial theory is based on the assumption of an efficient market that investors are completely rational and asset prices completely reflect information. Therefore, the price of assets depends on their basic value [4]. However, a series of empirical puzzles and market anomalies that cannot be explained by traditional financial theories have emerged in the financial market, such as the value effect [5], the scale effect [6], and the reversal effect [7], momentum effect [8], disposal effect [9] and so on. In the 1980s, Grossman and Stiglitz [3] established an information asymmetry model from the perspective of the existence of information costs, thus confirming that the efficient market hypothesis that prices perfectly reflect information does not exist. Moreover, some current studies have proposed explaining these anomalies from the perspective of investor sentiment from the perspective of behavioral finance theory. Therefore, our paper further explores their impact on financial markets from the perspective of information cost and investor sentiment.

First, Many theoretical and empirical studies have proven evidence that investor sentiment has an impact on financial markets. On the empirical side, a number of studies have shown that the existence and importance of investor sentiment is correct, and it has an important impact on stock prices, thereby leading to the difficulty of pricing [10–13]. Moreover, some studies also found that investor sentiment affects the stock market returns [14–17] and volatility [18, 19].

On the theoretical side, some asset pricing models have been developed to support the role of investor sentiment, such as Mendel and Shleifer [20], Yan [21], Xie et al. [22], Gao et al. [23], Yang and Gao [24], Yang and Zhang [25, 26], Yang and Li [27, 28] demonstrated this view. Mendel and Shleifer [20], Yan [21], Xie et al. [22] constructed asset pricing model that includes noise traders, their model illustrated the influence of noise on the stock price. For example, Mendel and Shleifer [20] presented a chase noise model that most rational but uninformed traders occasionally chase noise as if it were information, thereby amplifying the sentiment shocks on prices, leading to a small number of noise traders can have a great impact on market equilibrium disproportionate to their size in the market. Yan [21] presented a noise model, where individual biases often cannot be canceled out by aggregation. In addition, some studies integrated other factors into asset pricing models. Yang and Zhang [25, 26] constructed a sentiment asset pricing model with consumption, and showed that the stock price has a wealth-weighted average structure and the investor’s wealth proportion could amplify the sentiment shock on the asset price. Yang and Li [27, 28] integrated information into a sentiment asset pricing model, and found that the quality of information can enlarge the sentiment shocks on stock prices. Considering the limited arbitrage in the futures market, Gao et al. [23] established a sentimental pricing model of multi-period trading, discussing how the equilibrium price changes when rational arbitrageurs, long-term and short-term sentimental investors all exist in the market. At the same time, it also proved the empirical conclusion of Yang and Gao [24]: the short-term sentimental effect is greater than the long-term sentimental effect in a calm situation. In particular, Ma et al. [29] provided a continuous-time pure exchange model to study the impact of benchmark incentives and the impact of divergence on asset price dynamics. The study found that differences of opinion prompted institutional investors to take opposite strategies to the stock market.

Second, some scholars have studied the relationship between information costs and financial markets. For example, Grossman and Stiglitz [3] showed that because the existence of information cost, the traditional efficient market hypothesis does not exist, and the ratio of the expected effect of the informed investor to the rational but uninformed investor increases as the cost of information increases. However, in our model, the ratio of expected utility of informed institutional investors (informed) to rational but uninformed institutional investors (rational but uninformed investors) decreases first and then increases with the increase of information cost. Han and Yang [30] constructed a rational expectation equilibrium model, the study found that the precision of information is affected by the cost of information, but has nothing to do with network connectivity.

Although the current researches on these two types of literature have been very rich, there are still gaps in the research based on how information cost and investor sentiment jointly affect market equilibrium.

Therefore, based on the research framework of Grossman and Stiglitz, we construct an asset pricing model about information cost and investor sentiment. In our model, three types of market participants are involved. Among them, rational investors include informed institutional investors and rational but uninformed institutional investors. Informed institutional investors are those few investors who can observe extra information of assets by spending information cost; rational but uninformed institutional investors are those few investors who are affected by sentiment, they can only know the fundamental information of assets by observing prices. Rational but uninformed institutional investors can transform into informed institutional investors by choosing to spend information cost. Retail investors refer to the majority of investors who do not learn from prices and have a biased belief about the fundamental value of the asset, and they trade based solely on their sentiment. We analyze how the cost of information affects the composition of investors in the market, and find that investors are more inclined to be uninformed at the stage of lower information cost. In addition, our model mainly analyzes the impact of transactions lead by retail traders on market equilibrium.

This study has three main contributions to the existing literature. First, compared with previous studies, our model analyzes the impact of information cost C on the expected utility ratio of informed and rational but uninformed institutional investors. Partly consistent with the results of Grossman and Stiglitz [3] (1980)—the increase in information cost will increase the expected utility ratio of the insider to the uninformed. However, after considering the impact of the Internet on the cost of information and information propagation, at the stage when the cost of information is relatively small, the ratio of the expected utility of informed institutional investors to rational but uninformed institutional investors is decreasing, and thus analyze the information cost how to influence the proportional relationship between informed and rational but uninformed institutional investors. Second, our model incorporates two different investor sentiments into the asset pricing model, focusing on analyzing how retail investor sentiment affects the equilibrium price of assets under the background of rational but uninformed institutional investor sentiment is pessimism. Past studies have often shown that institutional investors or insider trading determine asset prices, but our research finds that the unanimous beliefs of retail investors can lead to a significant increase in asset prices. Finally, we set out from a new perspective—the retail investors clique—analyzing how retail investors make investors’ beliefs unanimous under the impact of the Internet and other information media, and thereby affect the equilibrium price of assets. It further enriches the research on investor sentiment and asset pricing.

The rest of paper is organized as follows. In Section 2, we set the economic environment and solve the model. Section 3 analyzes the impact of retail investors on asset prices. Section 4 considers the measurement of market stability and efficiency in addition to the sensitivity of market prices to investor sentiment. Section 5 draws a conclusion. All proofs and derivations are in the appendices.

## 2. Economic environment

This model is a static asset pricing model that contains one trading period. There are two tradable assets in the economy: one is the risk-free asset *M*_*i*_, it’s supply is elastic, and the return is R. The other is the risky asset (stock) *X*_*i*_, it’s supply quantity is 1, the price is P, and the asset value is uncertain. It will be realized at the end of the economic period, denoted by V. Therefore, the budget constraint *W*_0*i*_ of the i-th investor is:

$$\text{P}X_{i}+M_{i}=W_{0i}\equiv \bar{M_{i}}+P\bar{X_{i}}$$

Consistent with Grossman and Stiglitz [3], Mendel and Shleifer [20] to construct the value method of risky assets, we divide the fundamental value of risky assets into three terms: the first term is the unconditional expectation of risky assets is μ, and the second term is the extra information θ which is normally distributed with mean zero and variance $\sigma_{\theta }^{2}$ that investors can observe by spending a certain information cost C; the third term is a random disturbance term ε which is also normally distributed with mean zero and variance $\sigma_{\varepsilon }^{2}$ and independent of θ. Therefore, the fundamental value of risk assets is then given by:

V=μ+θ+ε (1)

There are three types of investors participating in this market: a few *λ*_*I*_ of informed institutional investors, a few *λ*_*O*_ of rational but uninformed institutional investors, and a mass *λ*_*N*_ of retail investors. Informed institutional investors choose to spend a certain information cost C to purchase information θ and to conduct transactions based on it, which means the informed institutional investors are completely rational; rational but uninformed institutional investors choose not to spend information cost, they learn by observing prices, and they trade under the influence of sentiment; retail investors conduct irrational transactions based on personal sentiment.

In this economic environment, the perception of risky assets by rational but uninformed institutional investors and retail investors includes their individual beliefs—investor sentiment *S*_*O*_ and *S*_*N*_. The sentiment of rational but uninformed institutional investors is pessimistic, denoted by a constant *S*_*O*_, the retail investors’ sentiment is optimistic, recorded as *S*_*N*_, and *S*_*N*_ is distributed normally with mean zero and variance $\sigma_{S_{N}}^{2}$. Moreover, θ, ε, and *S*_*N*_ are independent of each other.

We use MATLAB software to simulate the expected utility ratio of informed investors and rational but uninformed investors under different institutional sentiment, as well as the expected utility ratio of informed investors and retail investors, to judge the structure of investors in the market.

**Fig 2:**
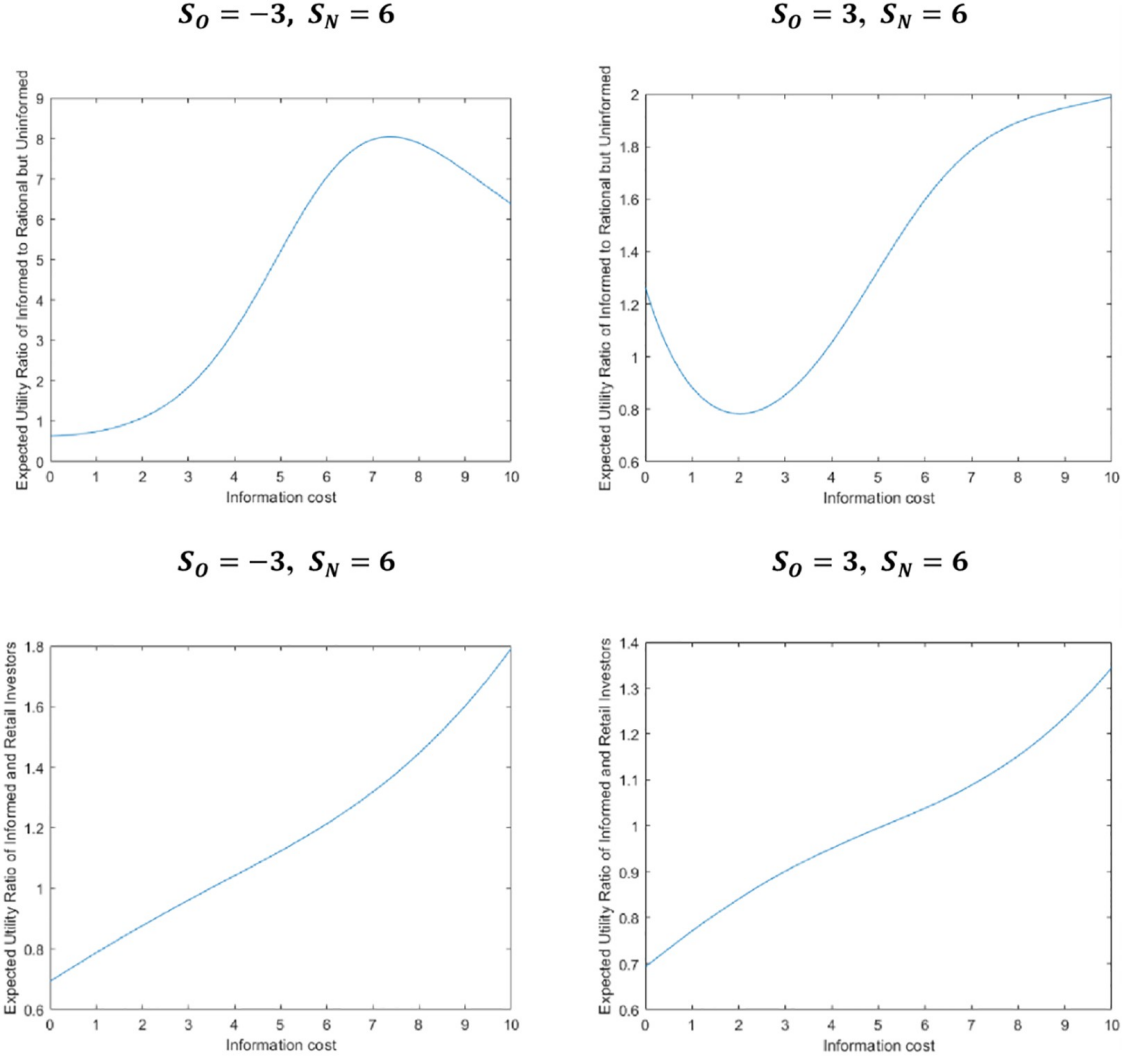
The expected utility ratio of different investors under different sentiment.

Fig 2 shows that the different sentiments of institutional investors have different effects on the financial market (as shown in Fig 2), that is, when the sentiments of institutional investors are different, the corresponding expected utility ratios of each type of investors are different, and the market results is also different. This article is based on the background of the game stop event, denote the pessimism of uninformed institutional investors with *S*_*N*_ = −3.

**Fig 3:**
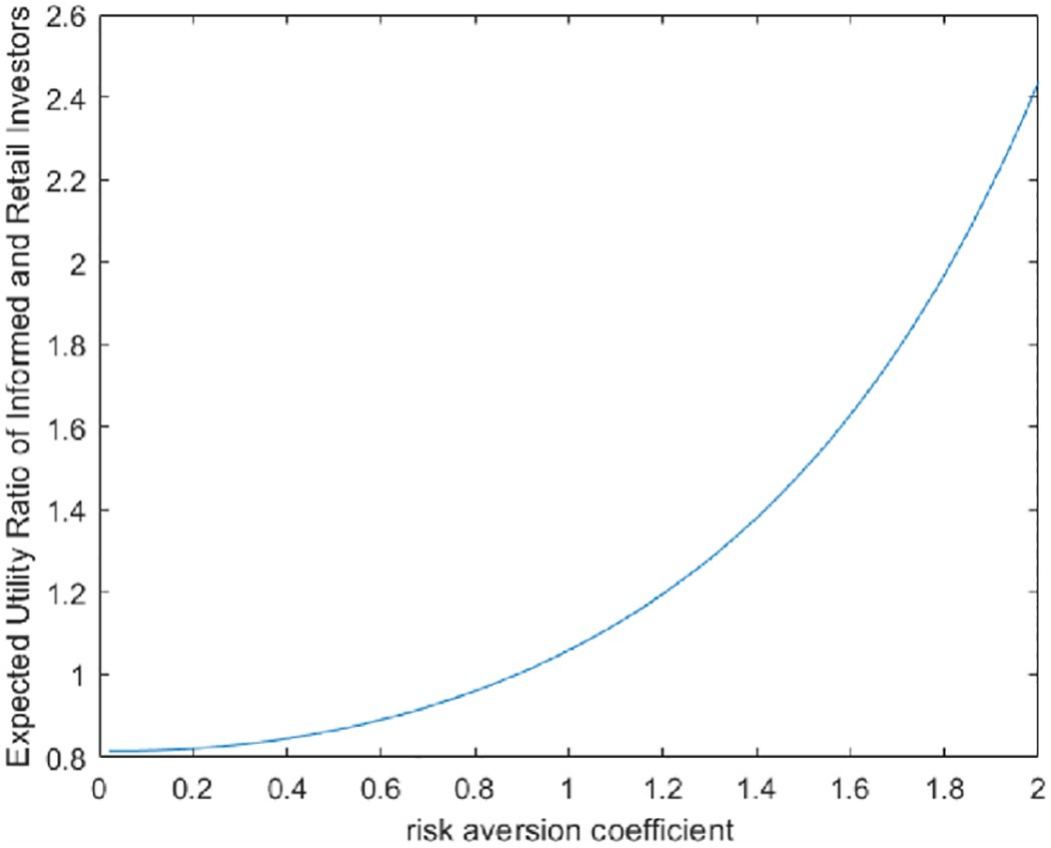
The relationship between the risk aversion coefficient and the expected effect ratio of insiders and retail investors.

We also draw the market equilibrium under different coefficients of risk aversion, and the graph shows that different coefficients of risk aversion lead to different choices of investors (see Fig 3). When the risk aversion coefficient is low, it also means that investors believe that the market conditions are better, and investors are more inclined to become retail investors. For the convenience of research, we consider the background of the article and assume that the risk aversion coefficient of each investor is the same.

Referring to the assumption in Grossman and Stiglitz [3], we assume that each investor has the same CARA utility function as:

$$\text{U}\left(W_{1i}\right)=-e^{-aW_{1i}},\;a>0$$ (2)

Where *a* denotes the absolute risk aversion coefficient, wealth *W*_1*i*_ obeys a normal distribution, and the budget constraint of each type of investor is *W*_0*i*_. Investors choose their own demand for risk assets *X*_*i*_ to maximize the expected personal utility, the maximum utility is:

$$\text{Max}\;\text{E}\left[\text{U}\left(W_{1i}\right)\right]\Leftrightarrow \text{Max}\left[E\left(W_{1i}\right)-\frac{1}{2}aVar\left(W_{1i}\right)\right]$$ (3)

Further available,

$$E\left(W_{1i}\right)-\frac{1}{2}aVar\left(W_{1i}\right)$$

$$=E\left(X_{i}V+\left(W_{0i}-X_{i}P-C\right)R-\frac{1}{2}aVar\left(X_{i}V+\left(W_{0i}-X_{i}P-C\right)R\right)\right)$$ (4)

Taking the first derivative of *X*_*i*_ from the above formula and setting it equal to 0, we can solve that the demand for risk assets of each type of investor:

$$X_{i}=\frac{E_{i}\left(V\right)-PR}{aVar_{i}\left(V\right)}$$ (5)

Furthermore, we can find the expected utility of informed institutional investors and the expected utility of rational but uninformed institutional investors. Since the ratio of the two types of investors is determined by whether they will spend the information cost C, when the expected utility of the former is greater than the latter, some investors will change from rational but uninformed institutional investors to informed institutional investors; and vice versa. Therefore, the overall equilibrium of the market requires they have same expected utility, which is:

$$\text{E}\left[\text{U}\left(W_{1i}^{I}\right)\right]/E\left[\text{U}\left(W_{1i}^{O}\right)\right]=1$$

Here, we also consider and analyze the influence of investor sentiment on several market statistics. First is sentiment sensitivity analysis; second is information sensitivity analysis; and the last is the measurement of market stability and efficiency in addition to the impact of sentiment on prices.

## 3. Economic equilibrium

The market we studied includes informed institutional investors, rational but uninformed institutional investors, and retail investors. This is similar to the economic environment of Mendel and Shleifer [20], but retail investors in our economy, also called noise traders in Mendel and Shleifer, account for the vast majority of the market. First, we set the market equilibrium price as P. At this time, the conditional expectation and conditional variance of the fundamental value of risk assets by informed institutional investors are as follows:

$$E_{i}^{I}\left(V\right)=\mu +\theta ,\;\;Var_{i}^{I}\left(V\right)=\sigma_{\varepsilon }^{2}$$

Similarly, the conditional expectation and conditional variance of the fundamental value of risky assets by rational but uninformed institutional investors are:

$$E_{i}^{O}\left(V\right)=\mu +E\left(\theta |P\right)+S_{O},\;Var_{i}^{O}\left(V\right)=\sigma_{O}^{2}=Var\left(\theta |P\right)+\sigma_{\varepsilon }^{2}$$

Where,

$$E\left(\theta |P\right)=\frac{\frac{\lambda_{I}}{\sigma_{\varepsilon }^{2}}\sigma_{\theta }^{2}}{{\left(\frac{\lambda_{I}}{\sigma_{\varepsilon }^{2}}\right)}^{2}\sigma_{\theta }^{2}+{\left(\frac{\lambda_{N}}{\sigma_{N}^{2}}\right)}^{2}\sigma_{SN}^{2}+\frac{\lambda_{o}\lambda_{I}}{\sigma_{O}^{2}\sigma_{\varepsilon }^{2}}\sigma_{\theta }^{2}}$$

$$\cdot \left\{PR\left[\frac{\lambda_{I}}{\sigma_{\varepsilon }^{2}}+\frac{\lambda_{O}}{\sigma_{O}^{2}}+\frac{\lambda_{N}}{\sigma_{N}^{2}}\right]-\mu \left[\frac{\lambda_{I}}{\sigma_{\varepsilon }^{2}}+\frac{\lambda_{O}}{\sigma_{O}^{2}}+\frac{\lambda_{N}}{\sigma_{N}^{2}}\right]+a-\frac{O}{\sigma_{O}^{2}}\cdot S_{O}\right\}$$ (6)

$$\sigma_{O}^{2}=\sigma_{\varepsilon }^{2}+\sigma_{\theta }^{2}\cdot \frac{\lambda_{N}{}^{2}\sigma_{SN}^{2}\sigma_{\varepsilon }^{4}}{\lambda_{I}{}^{2}\sigma_{\theta }^{2}\sigma_{N}^{4}+\lambda_{N}{}^{2}\sigma_{SN}^{2}\sigma_{\varepsilon }^{4}}$$ (7)

The conditional expectations and conditional variances of retail investors on the fundamental value of risky assets are:

$$E_{i}^{N}\left(V\right)=\mu +S_{N},\;Var_{i}^{N}\left(V\right)=\sigma_{N}^{2}=\sigma_{\theta }^{2}+\sigma_{\varepsilon }^{2}$$

With all the known results above, according to the market clearing conditions, we can get:

$$\frac{\mu +\theta -PR}{a\sigma_{\varepsilon }^{2}}\times \lambda_{I}+\frac{\mu +E\left(\theta |P\right)+S_{O}-PR}{a\sigma_{O}^{2}}\times \lambda_{o}+\frac{\mu +S_{N}-PR}{a\sigma_{N}^{2}}\times \lambda_{N}=1$$ (8)

Plugging this back into the market clearing equation and solving for the equilibrium price gives:

$$\text{P}=R^{-1}\left(\mu -A^{-1}+\frac{O}{Aa\sigma_{O}^{2}}\cdot S_{O}\right)+\frac{\lambda_{I}}{\text{AB}Ra\sigma_{\varepsilon }^{2}}\theta +\frac{\lambda_{N}}{\text{AB}Ra\sigma_{N}^{2}}S_{N}$$ (9)

Where we have defined A and B as:

$$A=\frac{\lambda_{I}}{a\sigma_{\varepsilon }^{2}}+\frac{\lambda_{O}}{a\sigma_{O}^{2}}+\frac{\lambda_{N}}{a\sigma_{N}^{2}}$$ (10)

$$B=1-\frac{\frac{\lambda_{O}\lambda_{I}}{\sigma_{O}^{2}\sigma_{\varepsilon }^{2}}\sigma_{\theta }^{2}}{{\left(\frac{\lambda_{I}}{\sigma_{\varepsilon }^{2}}\right)}^{2}\sigma_{\theta }^{2}+{\left(\frac{\lambda_{N}}{\sigma_{N}^{2}}\right)}^{2}\sigma_{S_{N}}^{2}+\frac{\lambda_{o}\lambda_{I}}{\sigma_{O}^{2}\sigma_{\varepsilon }^{2}}\sigma_{\theta }^{2}}$$ (11)

In (9), *R*^−1^ appears in each term because it is the risk discount factor. A is a factor describing the aggregate risk-bearing capacity of the market, the inverse of which corresponds to the risk-premium agents demand in equilibrium in the first term. $\frac{O}{Aa\sigma_{O}^{2}}\cdot S_{O}$ represents the effect of rational but uninformed institutional investors on asset prices. The second term describes the impact of the total information of the basic value on asset prices. The third term represents the impact of retail investor sentiment on market prices.

Eq (9) fully reflects all available information derived from the value and the noise, and supports the argument of De Long [31]. It shows that the mispricing of risk assets comes from two factors: on the one hand, it is the limited arbitrage ability of rational investors; on the other hand, it is the wrong perception of irrational investors.

According to Grossman and Stiglitz [3], the overall equilibrium condition of the information market is: the expected utility of each type of investor is equal, the relationship is expressed as:

$$\text{E}\left[\text{U}\left(W_{1i}^{I}\right)\right]/E\left[\text{U}\left(W_{1i}^{O}\right)\right]=1$$ (12)

$$\text{E}\left[\text{U}\left(W_{1i}^{I}\right)\right]/E\left[\text{U}\left(W_{1i}^{N}\right)\right]=1$$ (13)

We have solved the expected utility of informed institutional investors as:

$$\text{E}\left[U\left(W_{1i}^{I}\right)\right]=-exp\left[-a\left\{\left(W_{0i}^{I}-C\right)R+\frac{{\left(\mu +\theta -PR\right)}^{2}}{2a\sigma_{\varepsilon }^{2}}\right\}\right]$$ (14)

The expected utility of rational but uninformed institutional investors is:

$$\text{E}\left[U\left(W_{1i}^{O}\right)\right]=-exp\left[-a\left\{W_{1i}^{O}R+\frac{{\left(\mu +E\left(\theta |P\right)+S_{O}-PR\right)}^{2}}{2a\sigma_{O}^{2}}\right\}\right]$$ (15)

The expected utility of retail investors is:

$$\text{E}\left[U\left(W_{1i}^{N}\right)\right]=-exp\left[-a\left\{W_{1i}^{N}R+\frac{{\left(\mu +S_{N}-PR\right)}^{2}}{2a\sigma_{N}^{2}}\right\}\right]$$ (16)

**Fig 4:**
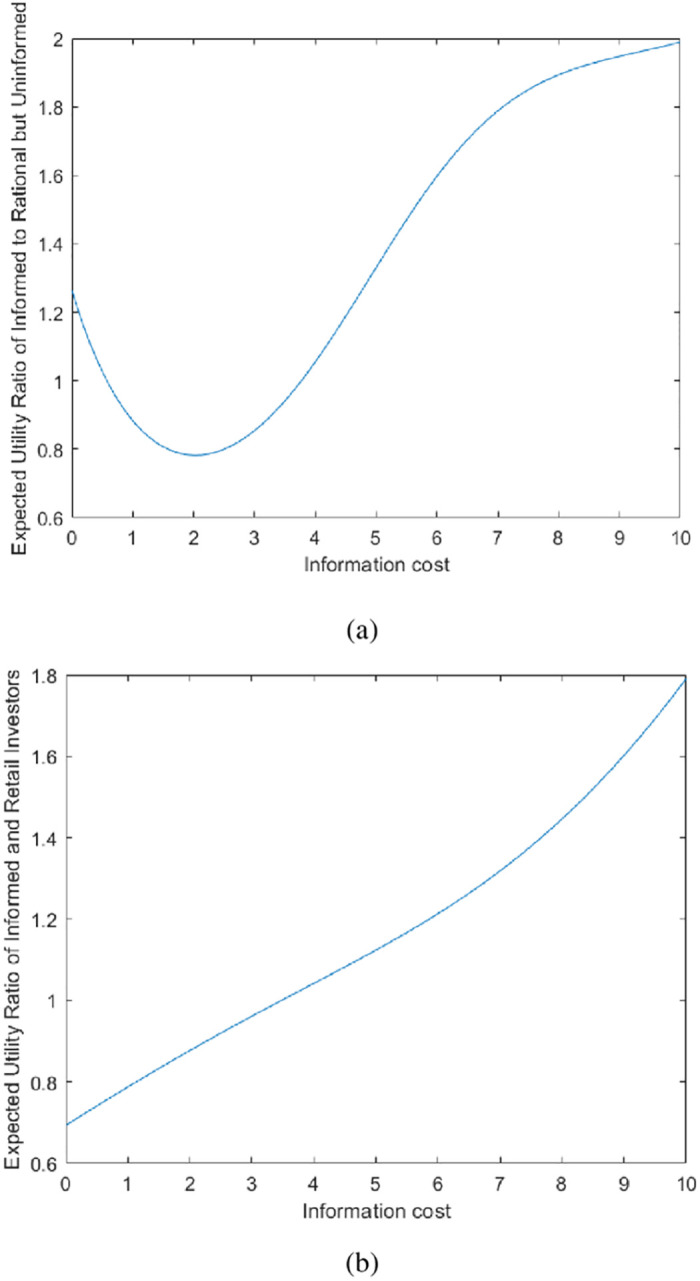
(a) A simulation diagram of the relationship between the expected utility ratio of informed institutional investors and rational but uninformed institutional investors and the cost of information. (b) A simulation diagram of the relationship between the expected utility ratio of informed institutional investors and retail investors and the cost of information.

We use MATLAB software to program and simulate the (12), (13) based on the degree of market information. As mentioned in the previous discussion (see Fig 2), we take *S*_*O*_ = −3, *S*_*N*_ = 6, and because retail investors’ beliefs tend to be the same under the influence of the internet, therefore the sentiment variation is small, so we take $\sigma_{S_{N}}^{2}=0.1$. In addition, based on the actual situation, we take other parameters as follows: $R=1.02,\;a=1.25,\;\sigma_{\varepsilon }^{2}=1,\;\sigma_{\theta }^{2}=1$. The results of the numerical simulation are as follows:

Fig 4 shows that investors are more likely to be uninformed at a stage of lower information costs. In particularly, the ratio of expected utility between informed institutional investors and rational but uninformed institutional investors decreases first and then increases with the increase of information cost. However, the Grossman and Stiglitz [3] study found that the ratio of the expected utility of the insider to the uninformed person increases with the increase of the information cost. In our model, the ratio of the expected utility of informed institutional investors to rational but uninformed institutional investors decreases when the information cost is low, and increases when the information cost increases to a certain extent.

Due to the development of the Internet, people can often get a lot of information through communication with each other. Therefore, retail investors affected by sentiment decide their trading direction based on the relevant information they get. At the same time, institutional investors have insight into the investment psychology and trading behavior of retail investors, and a large number of retail investors have sufficient funds to promote asset prices. Therefore, rational but uninformed institutional investors decide to become informed institutional investors, and even follow retail investors to trade for profit. In Fig 4, it is shown that the ratio of the expected utility of the two types of investors is reduced at the stage of lower information cost.

When the information cost increases to a certain point, due to lack of financial support, retail investors have a weaker ability to obtain relevant information. Therefore, the relevance of transactions is weak and transactions are more scattered. In this case, some rational but uninformed institutional investors are willing to spend a certain cost to obtain relevant information in order to obtain benefits, as described in Fig 4: in the stage of higher information cost, the ratio of the expected utility of the two types of investors is increased. This is consistent with the findings of Grossman and Stiglitz [3].

According to the above analysis, we can get the following proposition.

**Proposition 1**: When institutional investors are short on a certain stock and retail investors’ sentiment variation is small, the ratio of expected utility between informed institutional investors and rational but uninformed institutional investors decrease at the stage of lower information cost.

Through the above analysis, we learn that when the information cost is small, most investors will not spend the cost to purchase information. In the next part, we will analyze the sensitivity of prices to retail investor sentiment under different market composition.

## 4. Price sensitivity to retail investor sentiment

What we are interested is whether it is possible for ∂*P*/∂*S*_*N*_ to be large. It describes how asset prices deviate from their rational expected value under the influence of retail investor sentimental shocks. From the equilibrium price Eq (9), the specific expression of the sentimental sensitivity coefficient can be given as:

$$\frac{\partial P}{\partial S_{N}}=\frac{\lambda_{N}}{\left(\frac{\lambda_{I}}{a\sigma_{\varepsilon }^{2}}+\frac{\lambda_{O}}{a\sigma_{O}^{2}}+\frac{\lambda_{N}}{a\sigma_{N}^{2}}\right)\left(1-\frac{\frac{\lambda_{O}\lambda_{I}}{\sigma_{O}^{2}\sigma_{\varepsilon }^{2}}\sigma_{\theta }^{2}}{{\left(\frac{\lambda_{I}}{\sigma_{\varepsilon }^{2}}\right)}^{2}\sigma_{\theta }^{2}+{\left(\frac{\lambda_{N}}{\sigma_{N}^{2}}\right)}^{2}\sigma_{S_{N}}^{2}+\frac{\lambda_{o}\lambda_{I}}{\sigma_{O}^{2}\sigma_{\varepsilon }^{2}}\sigma_{\theta }^{2}}\right)Ra\sigma_{N}^{2}}$$ (17)

**Fig 5:**
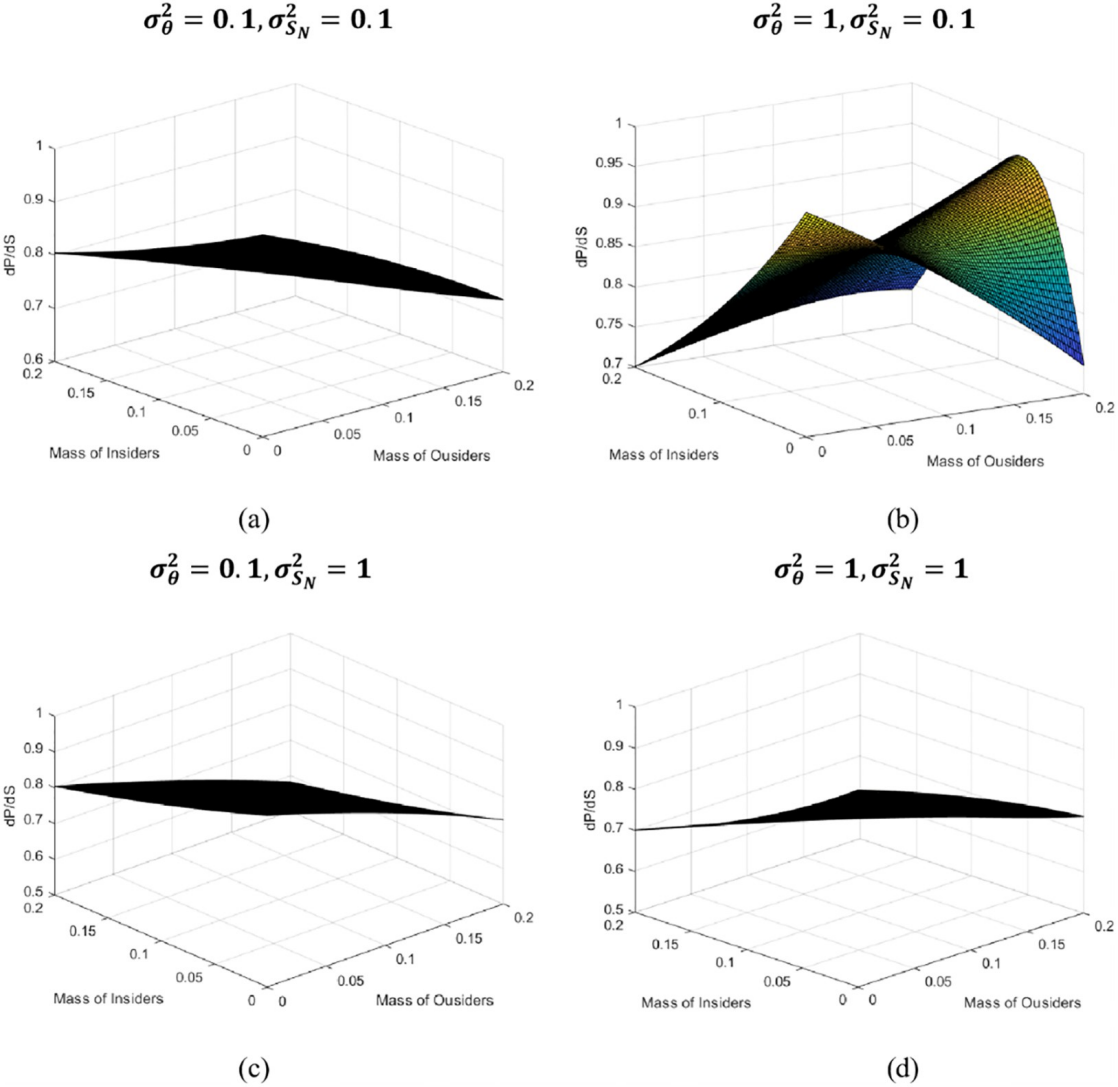
Sensitivity analysis of price to retail investor sentiment.

In order to quantitatively analyze the influence of investor sentiment on the equilibrium price, we give a numerical simulation of the ∂*P*/∂*S*_*N*_ under different information quality and degree of sentimental variation. Therefore, in the numerical example, the value range of informed institutional investors and rational but uninformed institutional investors is set to [0,0.2], taking into account the sentimental sensitivity coefficient and the average quality of information of informed institutional investors $\sigma_{\theta }^{2}$ is directly related to the average size of retail investor sentiment $\sigma_{S_{N}}^{2}$. Here we analyze four situations, that is (${\sigma_{\theta }^{2}=0.1,\;\sigma }_{S_{N}}^{2}=0.1),\;({\sigma_{\theta }^{2}=1,\;\sigma }_{S_{N}}^{2}=0.1),\;({\sigma_{\theta }^{2}=0.1,\;\sigma }_{S_{N}}^{2}=1$) and (${\sigma_{\theta }^{2}=1,\;\sigma }_{S_{N}}^{2}=1$). Set the parameter value according to the actual situation, given R = 1.02, *a* = 1.25, μ = 20, *S*_*O*_ = −3. For the four combinations of parameters $\sigma_{\theta }^{2}$ and ${\;\sigma }_{S_{N}}^{2}$, the numerical examples of the sentimental sensitivity coefficient in the range of *λ*_*I*_ ∈ [0,0.2] and *λ*_*O*_ ∈ [0,0.2] are shown in Tables 1–4. Correspondingly, the numerical simulations obtained by using MATLAB software are shown in Fig 5(a)–5(d).

**Table 1:** Price sensitivity to retail investor sentiment ∂P/∂SN when the information quality is low and the sentimental variation is small (${\sigma_{\theta }^{2}=0.1,\;\sigma }_{S_{N}}^{2}=0.1$).

*λ*_*I*_	*λ*_*O*_	0.01	0.05	0.1	0.2
0	0	0.01	0.05	0.1	0.2
0.01	0	0.9804	0.9304	0.8727	0.7353
0.05	0	0.9308	0.8838	0.8306	0.7095
0.1	0	0.8841	0.8383	0.7913	0.6848
0.2	0	0.8036	0.7649	0.7232	0.6381

**Table 2:** Price sensitivity to retail investor sentiment ∂P/∂SN when the information quality is high and the sentimental variation is small (${\sigma_{\theta }^{2}=1,\;\sigma }_{S_{N}}^{2}=0.1$).

*λ*_*I*_	*λ*_*O*_	0.01	0.05	0.1	0.2
0	0	0.01	0.05	0.1	0.2
0.01	0	0.9804	0.9304	0.8727	0.7353
0.05	0	0.8937	0.8882	0.8914	0.9440
0.1	0	0.8184	0.8364	0.8644	0.9426
0.2	0	0.7003	0.7209	0.7390	0.7429

**Table 3:** Price sensitivity to retail investor sentiment ∂P/∂SN when the information quality is low and the sentimental variation is large (${\sigma_{\theta }^{2}=0.1,\;\sigma }_{S_{N}}^{2}=1$).

*λ*_*I*_	*λ*_*O*_	0.01	0.05	0.1	0.2
0	0	0.01	0.05	0.1	0.2
0.01	0	0.9804	0.9304	0.8727	0.7353
0.05	0	0.9308	0.8813	0.8246	0.6914
0.1	0	0.8841	0.8354	0.7799	0.6511
0.2	0	0.8036	0.7567	0.7037	0.5835

**Table 4:** Price sensitivity to retail investor sentiment ∂P/∂SN when the information quality is high and the sentimental variation is large (${\sigma_{\theta }^{2}=1,\;\sigma }_{S_{N}}^{2}=1$).

*λ*_*I*_	*λ*_*O*_	0.01	0.05	0.1	0.2
0	0	0.01	0.05	0.1	0.2
0.01	0	0.9804	0.9304	0.8727	0.7353
0.05	0	0.8937	0.8490	0.7990	0.6887
0.1	0	0.8184	0.7783	0.7347	0.6442
0.2	0	0.7003	0.6671	0.6307	0.5623

Fig 5(a), 5(c) and 5(d) show that when the market proportion of retail investors tends to 1, the sensitivity of retail investor sentiment reaches its maximum. As the proportion of informed institutional investors increases, information is gradually integrated into asset prices in transactions, thus reducing the sensitivity of retail investor sentiment and increasing the sensitivity of prices to information (as shown in Fig 5). Similarly, with the increase of rational but uninformed institutional investors, some rational but uninformed institutional investors follow informed institutional investors to trade, thereby reducing the impact of retail investor sentiment on prices.

However, according to Fig 5(b), we find that in a market environment where the quality of information is high and the sentiment variation is small (${\sigma_{\theta }^{2}=1,\;\sigma }_{S_{N}}^{2}=0.1$), the sensitivity of retail investor sentiment reaches a local maximum of 0.9645 when *λ*_*O*_ = 0.2 and *λ*_*I*_ = 0.072. This market result stems from the situation that rational but uninformed institutional investors short a certain stock (*S*_*O*_ = −3), which lead to the decline in the stock price. On the one hand, with the promotion of information dissemination by the Internet, retail investors prefer the low-priced stocks, and concentrate ($\sigma_{S_{N}}^{2}=0.1$) on longing the stock, resulting in a substantial increase in the stock price due to the optimistic sentiment of retail investors (*S*_*N*_ = 6). On the other hand, as the positions hold by short sellers exceed the tradable stocks in the market, and retail investors are long the stock, resulting in a sharp rise in the price of the stock, the short sellers have to buy the stock in order to cover their positions, further driving up the price. Fig 5(b) graphically depicts this phenomenon, and provides part of the explanation for the unusually high stock price of Game Stop in early 2021 that retail investors cliqued and confronted institutional investors.

**Fig 6:**
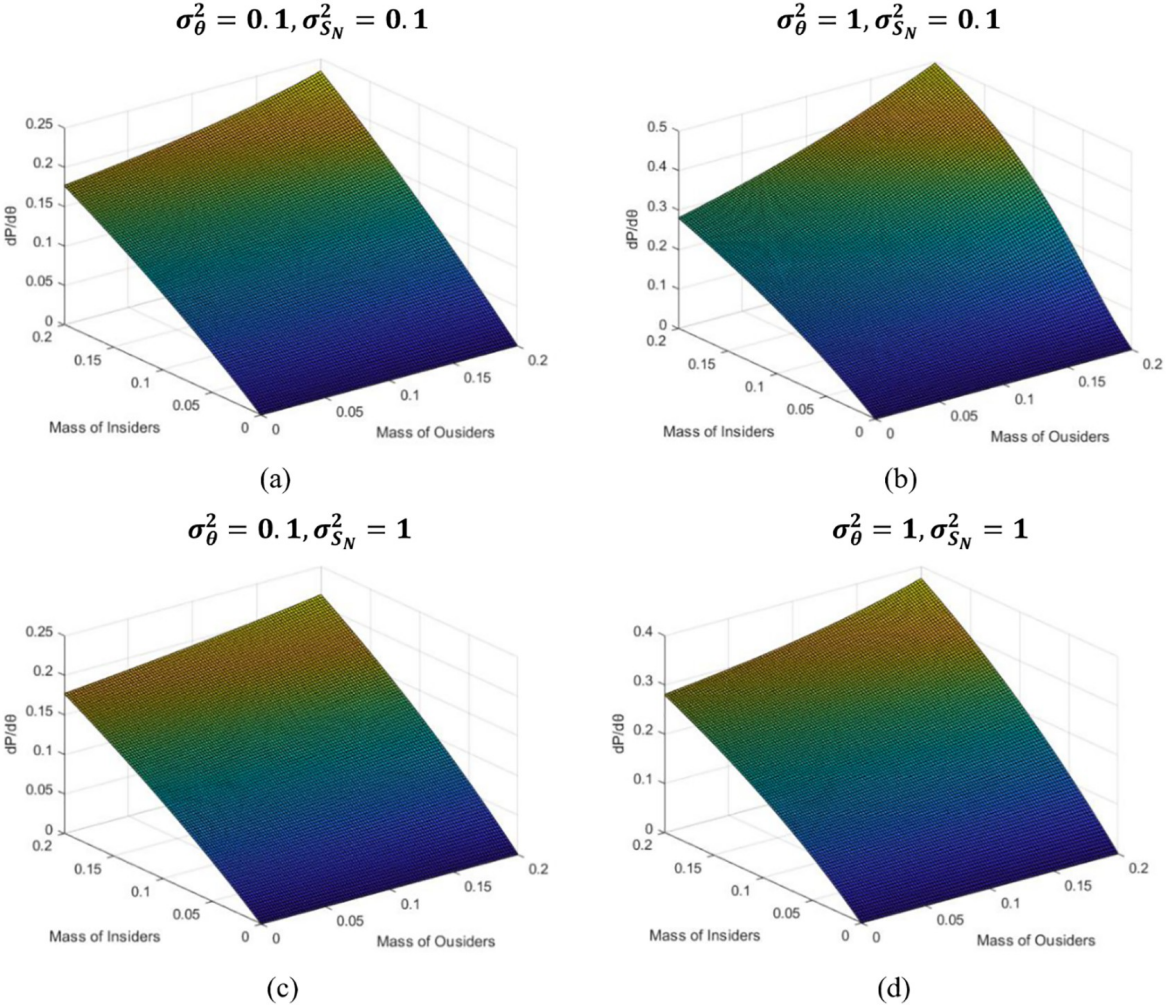
Information sensitivity analysis.

In addition, we also find that when the quality of information is high and retail investor sentiment is less variable, increasing institutional investors makes the degree of information integrated into the price more fully. In Fig 6(b), when the size of the retail investor’s transaction volume is reduced to 60% of the market, the information sensitivity reaches 0.5, which is larger than the value in other market environments. Considering that the Internet connects retail investors together, making them trade based on market noise increases their sentimental sensitivity. On the other hand, information is also transmitted to investors in the market through the Internet. When information about asset prices is released in the market, it encourages them to conduct transactions in the same direction and at the same time better integrate the information into the price.

Based on the above analysis, we get proposition 2.

**Proposition 2**: When there are three types of investors in the market: informed institutional investors, rational but uninformed institutional investors, and retail investors, the higher the quality of information ($\sigma_{\theta }^{2}=1$) and the lower variation of retail investors in the market (${\;\sigma }_{S_{N}}^{2}=0.1$), retail investors clique increase the sensitivity of prices to retail investor sentiment.

Through the above analysis, we find that the possibility of retail investor sentiment variation and scale of retail investors have a significant impact on asset prices. Therefore, in the next section, we will analyze in detail how the possibility of retail investor sentiment variation and scale of retail investors affect market efficiency.

## 5. Other measures of market efficiency and stability

### 5.1 Variance of equilibrium asset prices

Another metric to measure the impact retail investors have on market efficiency and stability is the variance of equilibrium asset prices. It can be written as:

$$\sigma_{P}^{2}={\left(\frac{\partial P}{\partial S_{N}}\right)}^{2}\sigma_{S_{N}}^{2}+{\left(\frac{\partial P}{\partial \theta }\right)}^{2}\sigma_{\theta }^{2}$$ (18)

It can be seen from Eq (18) that the variance of asset prices consists of two parts: the first part of the variance is caused by the sentimental impact of retail investors, which drives the price to gradually deviate from the basic value of the asset, called “bad variance”; the second part is the price variance caused by the integration of the information in the market into the asset price, called “good variance”. Its effect is the opposite of the variance in the first part, because it pushes the price closer to the fundamental value.

From the analysis of sentimental and information sensitivity in the previous part, we can learn that when the size of informed institutional investors and rational but uninformed institutional investors is sufficiently small, due to sentimental shock and information integration into the price, the “bad variance” and the “good variance” all increase. Therefore, the variance of the equilibrium price of the asset increases in the area of our concern.

Next, we will explore the impact of retail investor size on the variance of prices in the context of different levels of sentiment variation from both mathematical and image analysis. The scale of retail investors affects the price variance from two aspects: on the one hand, it has an impact on the price variance caused by sentimental shocks; on the other hand, it has an impact on the price variance caused by the integration of information into asset prices.

Finding the first-order derivative of the price variance to the retail investor’s part, we get:

$$\frac{\partial \sigma_{P}^{2}}{\partial \lambda_{N}}=\partial {\left(\frac{\partial P}{\partial \theta }\right)}^{2}\sigma_{\theta }^{2}/\partial \lambda_{N}+\partial {\left(\frac{\partial P}{\partial S_{N}}\right)}^{2}\sigma_{S_{N}}^{2}/\partial \lambda_{N}$$

$$=\frac{2\lambda_{I}\sigma_{\theta }^{2}}{ABRa\sigma_{\varepsilon }^{2}}\cdot \frac{\partial \left(\lambda_{I}/ABRa\sigma_{\varepsilon }^{2}\right)}{\partial \lambda_{N}}+\frac{2\lambda_{N}\sigma_{S_{N}}^{2}}{ABRa\sigma_{N}^{2}}\cdot \frac{\partial \left(\lambda_{N}/ABRa\sigma_{N}^{2}\right)}{\partial \lambda_{N}}$$ (19)

The first term on the right side of the formula (19) represents the impact of the size of retail investors on the sensitivity of information, and the second term represents the sensitivity of retail investors’ size to sentiment. Through further analysis, we easily get $\partial {\left(\frac{\partial P}{\partial \theta }\right)}^{2}\sigma_{\theta }^{2}/\partial \lambda_{N}<0$, and $\partial {\left(\frac{\partial P}{\partial S_{N}}\right)}^{2}\sigma_{S_{N}}^{2}/\partial \lambda_{N}>0$. Therefore, the influence of the variance of retail investor scale price is determined by the sensitivity of the retail investor’s size to sentiment and the sensitivity of information. When the size of retail investor’s sensitivity to sentiment exceeds the sensitivity of information, retail investor has a positive effect on the price variance. Similarly, when the size of retail investors is more sensitive to information than sentiment, retail investors have a negative impact on price variance.

In order to more intuitively analyze how the size of retail investors in the market affects the variance of asset prices, we choose *λ*_*I*_ ∈ [0,0.2] and *λ*_*O*_ ∈ [0,0.8] to control the range of retail investors’ market share of the market as [0,1]. Other parameters are set as follows: R = 1.02, $a=1.25,\;\mu =20,\;\sigma_{\theta }^{2}=0.1,\;\sigma_{\varepsilon }^{2}=1$.

**Fig 7:**
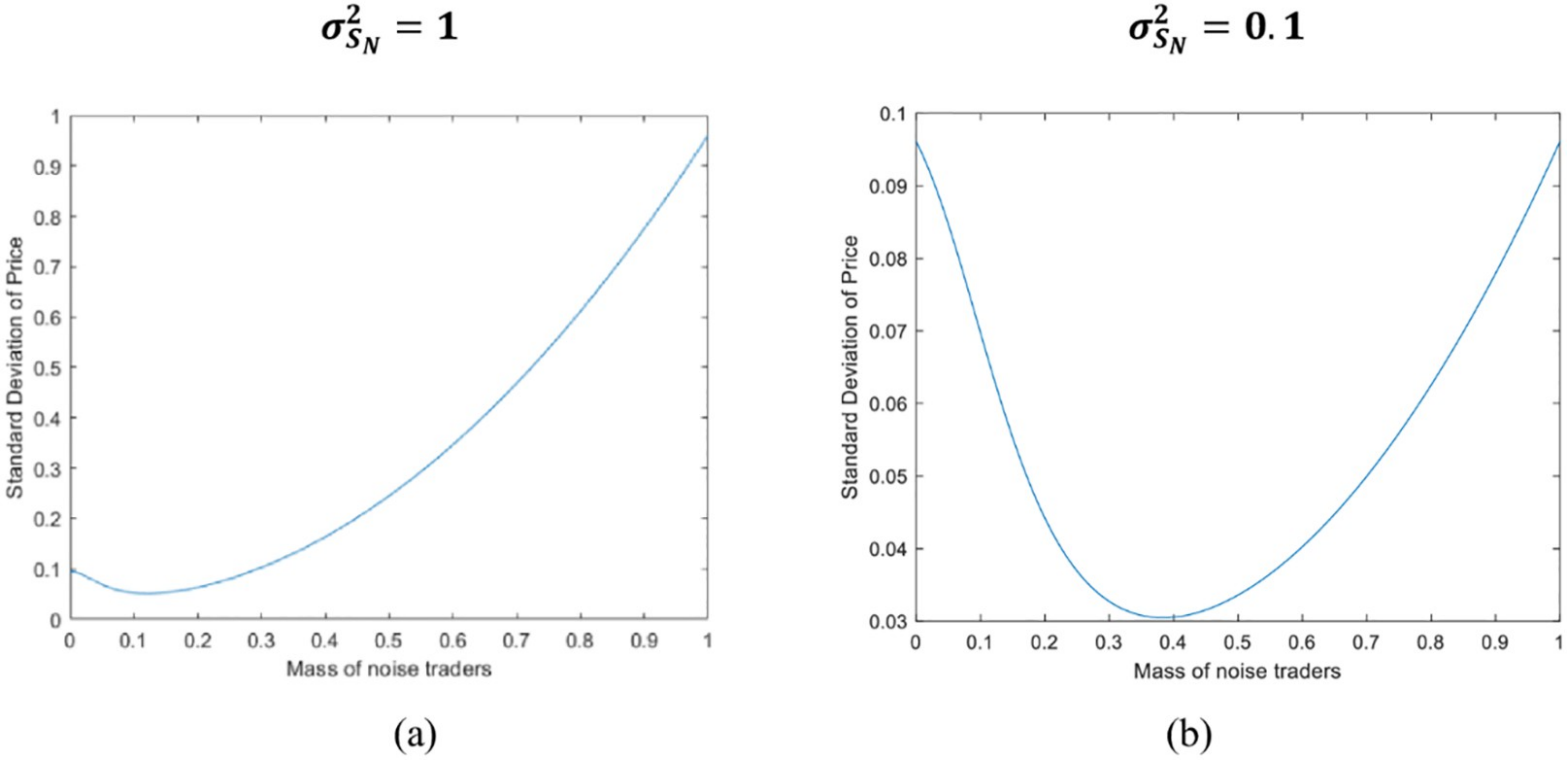
Standard deviation analysis of equilibrium price.

Fig 7(a) shows that in the case of large variation of retail investor sentiment (${\;\sigma }_{S_{N}}^{2}=1$), the variance of asset prices increases as the size of retail investors increases. This is because the market is relatively unstable at this time, and the expansion of the size of retail investors further enhances the instability of the financial market, thereby increasing the variance of asset prices.

Fig 7(b) shows that in the case of small variation of retail investor sentiment (${\;\sigma }_{S_{N}}^{2}=0.1$), the variance of asset prices first decreases and then increases as the size of retail investors increases. This is because the market is relatively calm at this time, and the number of informed institutional investors and retail investors is small. A large number of rational but uninformed investors mistakenly regard noise as information to chase, thereby amplifying the sentimental impact, and keep the price away from its basic value. Therefore, when retail investors tend to zero, the price variance is larger. With the increase of retail investors, rational but uninformed investors gradually distinguish information and noise more clearly, so that the price variance gradually decreases, this is consistent with what Mendel and Shleifer (2012) model explained. With the further increase in the scale of retail investors, investors’ demand for risky assets has increased, and market risks have also increased, causing asset prices to deviate from their true value.

Based on the above analysis, we sum up and get the following proposition.

**Proposition 3**: When the size of the retail investor’s impact on the “bad variance” exceeds its impact on the “good variance”, that is $\partial {\left(\frac{\partial P}{\partial S_{N}}\right)}^{2}\sigma_{S_{N}}^{2}/\partial \lambda_{N}>\partial {\left(\frac{\partial P}{\partial \theta }\right)}^{2}\sigma_{\theta }^{2}/\partial \lambda_{N}$, retail investors’ price variance increases.

**Proposition 4**: When investor sentiment variability is large (${\;\sigma }_{S_{N}}^{2}=1$), increase retail investors to increase the variance of price; when investor sentiment variability is small (${\;\sigma }_{S_{N}}^{2}=1$), the variance of asset prices first decreases and then increases with the increase of retail investors.

The above part focuses on the analysis of the impact of investor sentiment on asset prices and market efficiency. Next, we will analyze the impact of retail investors on market efficiency from another perspective.

### 5.2 Information level of the price system

Refer to the definition of "informative-ness of the price system" by Grossman and Stiglitz [3]—the square of the correlation coefficient between asset price P and information θ—(*corr*(*P*, *θ*))^2^, this metric measures the market’s reflection of institutional investor investment in terms of the performance of the information. Similarly, it also reflects the market’s reaction of retail investor investment in terms of the performance of the noise. We write its expansion as:

$${\left(corr\left(P,\theta \right)\right)}^{2}=\frac{1}{1+\frac{\lambda_{N}^{2}\sigma_{S_{N}}^{2}\sigma_{\varepsilon }^{4}}{\lambda_{I}^{2}\sigma_{N}^{4}\sigma_{\theta }^{2}}}$$ (20)

Obviously, the number of retail investor *λ*_*N*_ has an inverse relationship with the information degree of the price system, and the first-order derivative of the information degree for *λ*_*N*_ is:

$$\frac{\partial {\left(corr\left(P,\theta \right)\right)}^{2}}{\partial \lambda_{N}}=-\frac{\lambda_{I}^{2}\sigma_{N}^{4}\sigma_{\theta }^{2}\sigma_{\varepsilon }^{4}\sigma_{S_{N}}^{2}}{{\left(\lambda_{N}^{2}\sigma_{S_{N}}^{2}\sigma_{\varepsilon }^{4}+\lambda_{I}^{2}\sigma_{N}^{2}\sigma_{\theta }^{2}\right)}^{2}}$$ (21)

Its value is obviously negative.

By analyzing the Eq (21), it is not difficult to find that when $\sigma_{S_{N}}^{2}$ is large, the value of (∂(*corr*(*P*, *θ*))^2^)/(∂*λ*_*N*_) is small, and the curve appears to be steeper on the picture.

Under this situation, the financial market has shown its existing instability. When retail investors’ trading behavior becomes more aggressive, it further reduces the level of information available in the market. In other words, when retail investor sentiment variability is large (${\;\sigma }_{S_{N}}^{2}=1$), the increase of its size increases the noise trading volume in the market and pushes the price to further deviate from its true value.

**Fig 8:**
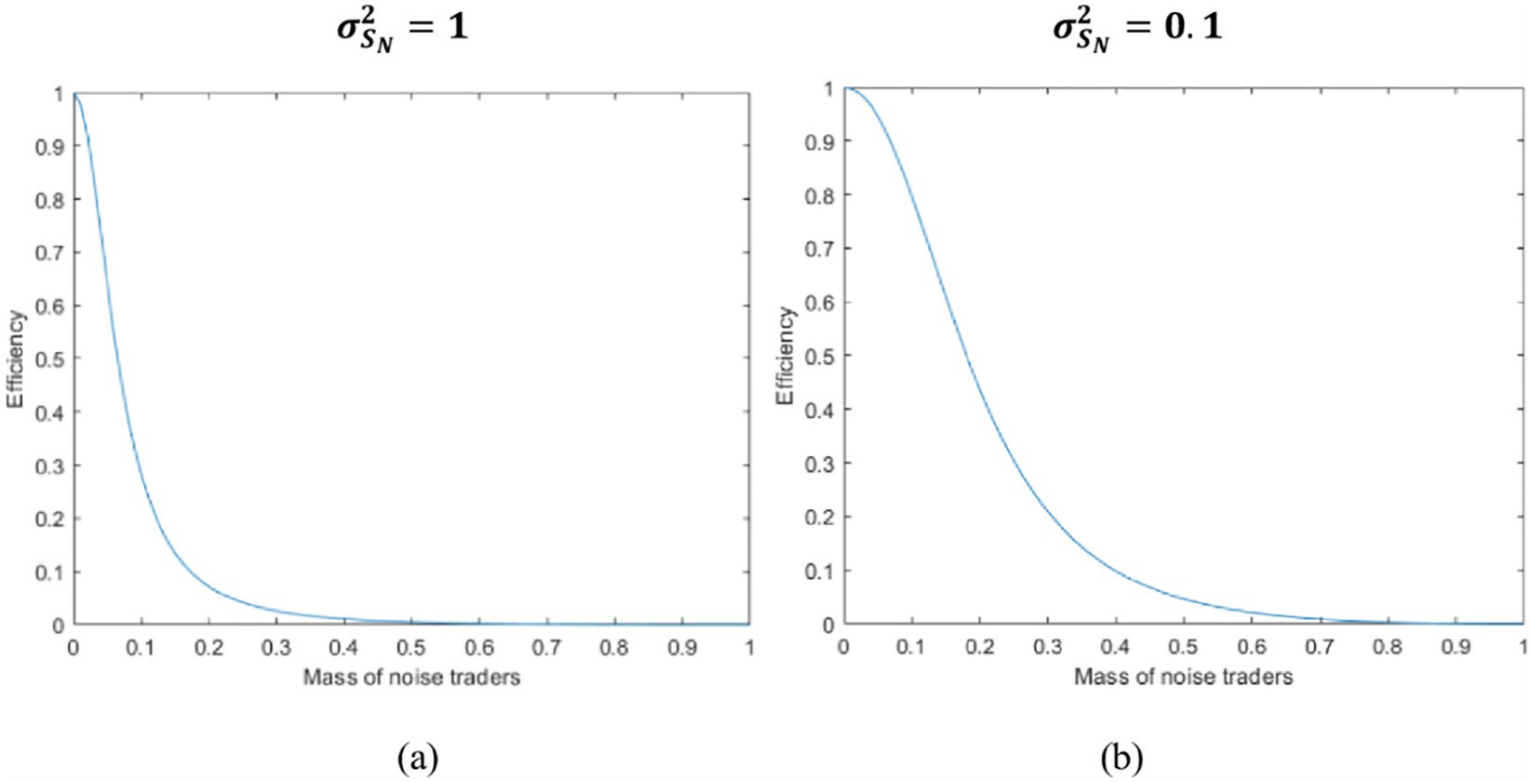
Correlation analysis of equilibrium price P and information θ.

In addition, we also obtained Fig 8 through numerical simulations, which confirmed this conclusion. Among them, the selection of relevant parameters is the same as in section 5.1.

From Eq (20) and Fig 8, we can easily obtain the following propositions:

**Proposition 5**: Increasing retail investors often reduces the informative-ness of the price system. In addition, when the sentimental variation of retail investors is large (that is, $\sigma_{S_{N}}^{2}$ is large), the informative-ness of the price system is more sensitive to changes in the market proportion of retail investors.

Investors as a group, especially for investors who lack financial support and are susceptible to sentiment, the psychological deviation between each other will not usually offset. In particular, under a specific market structure and information structure, the interaction between individuals leads everyone to make convergent decisions. Therefore, individual decisions will cause systematic deviations in the group, which will cause price to seriously deviate from the fundamental value of the asset.

## 6. Conclusion

Many financial anomalies cannot be explained by traditional financial theories, and financial markets are not always effective. When investors agree on the judgment of the stock market and adopt the same investment strategy, it may determine the trend of the market. Incorporating human psychology and behavior into financial pricing model, and according this to analyze investors’ investment strategies and market anomalies are of great significance for both institutional and retail investors’ investment decisions.

We have built a model, a key feature of the model is the low cost of obtaining information. At this time, when rational but uninformed institutional investors are bearish on a certain stock, and the variation of retail investor sentiment is small, a large number of retail investors clique to buy this stock centrally under the influence of the information network. First of all, in this model, retail investor sentiment variability is small, and the ratio of the expected utility of informed institutional investors to rational but uninformed institutional investors decreases first and then increases with the increase of information cost. The decrease stage is the area that we focus on under our research. In addition, a large number of retail investors clique to buy stocks shorted by institutional investors, thereby using the power of the group to drive up the price of the stock, rather than making profit by shorting a stock as institutional investors expected. This model provides part of the explanation for the unusually high stock price of Game Stop in early 2021 that retail investors cliqued and confronted institutional investors. Secondly, when retail investor sentiment variability is small, the variance of asset prices appears to have a maximum value when the number of retail investors tends to zero (see Mendel and Shleifer [20]) and then declines, and then increase with retail traders increase as their share of the market increases. Finally, we find that in the case of large investor sentiment variability, the information degree of the price system is more sensitive to the changes of the market proportion of retail investors.

In 2021, this socially concerned incident of confrontation between retail investors and institutions reflects the contradictions in global politics, economy, society and other aspects. However, in this paper, we mainly analyze the reasons behind this event from the perspective of retail investor sentiment. At the same time, this kind of event brings long-term influence and important enlightenment on the operation law of the financial market and system construction. We hope to analyze the causes of such events from multiple perspectives and explore the corresponding regulatory measures in the future theoretical and empirical research.

## Supporting information

S1 Data (CSV)

S1 File (TXT)
